# Poisoning from Pennyroyal

**Published:** 1903-10

**Authors:** 


### POISONING FROM PENNYROYAL.

HOLLAND, (*Virginia Medical Semi-monthly*, October 24, 1902), reports a case occurring in a woman, who upon advice of a physician took oil of pennyroyal as an abortifacient. Seven drops were twice taken in the course of three hours, and three hours later a half teaspoonful. The woman went to bed and to sleep, awakening in about an hour complaining of nausea and dizziness.

She vomited and almost immediately was seized with a violent convulsion. The spasm lasted only a few minutes and left the patient tossing about and talking incoherently. Consciousness was regained in about half an hour. There was great weakness, a rapid, feeble pulse, cramps in the stomach and a slight desire to go to stool. Recovery was complete, but the flow did not come on.

